# The complete mitochondrial genome of *Laevistrombus canarium* (Gastropoda: Stromboidae)

**DOI:** 10.1080/23802359.2021.1875920

**Published:** 2021-02-12

**Authors:** Hung-Tai Lee, Cheng-Hsin Liao, Chang-Wen Huang, Yung-Cheng Chang, Te-Hua Hsu

**Affiliations:** a Department of Environmental Biology and Fisheries Science, National Taiwan Ocean University, Keelung, Taiwan; b Department of Aquaculture, National Taiwan Ocean University, Keelung, Taiwan

**Keywords:** Dog conch, mitogenome, phylogenetic analysis

## Abstract

*Laevistrombus canarium* is a marine gastropod species with high economical value. The complete mitochondrial genome of *L. canarium* has been characterized in this study. The circular mitogenome is 15626 bp in length and comprises 13 protein-coding genes (PCGs), 22 transfer *RNA* genes, and two ribosomal RNA (*rRNA*) genes. The organization of these genes is consistent with that of other stromboidae species. The overall base composition of mitochondrial genome is 30.87% A, 38.99% T, 15.54% G, and 14.60% C, with 69.86% AT. Phylogenetic analysis further implies that *L. canarium* is placed within the Stromboidae.

The dog conch (*Laevistrombus canarium*) is a marine gastropod species that naturally inhabits the muddy and sandy bottoms (Cob et al. 2012). The geographic distribution of this species widely ranges from southern India to Melanesia, extending north to Japan and south to Australia (Poutiers 1998). The *L. canarium* is an economically important species with high market value since there is an increasing demand for this edible species as a seafood product. In this study, we aim to report the first complete mitochondrial genome of *L. canarium* and further analyze its phylogenetic position.

The specimen of *L. canarium* was collected from the coast water off Penghu island of Taiwan (119.5°E, 23.5°N) in March of 2020 and stored at National Taiwan Ocean University with a specimen number (NTOU-LC-01-2020). The total genomic DNA was prepared and then followed by the pair-end sequencing (2 × 150 bp) with Novaseq (Illumina, San Diego, CA). The *de novo* assembly of complete mitochondrial genome of *L. canarium* was performed using Geneious Prime version 2020.2 (Kearse et al. 2012). The identification and annotation of protein-coding genes (PCGs) were conducted using ORFfinder (https://www.ncbi.nlm.nih.gov/orfnder) with invertebrate mitochondrial genetic code. Additionally, the transfer RNA (tRNA) and ribosomal RNA (*rRNA*) genes were identified and annotated using MITOS Web Server (Bernt et al. 2013).

The complete mitochondrial genome of *L. canarium* is a typical closed-circular molecule with 15,626 bp in length (GenBank accession number: MT937083). The organization of mitochondrial genomes of *L. canarium* is consistent with that of other Stromboidae species (Jiang et al. 2019). It contains 13 PCGs, 22 tRNAs, and two ribosomal RNA (12S rRNA and 16S rRNA). The overall base composition of mitochondrial genome is biased toward A + T content at 69.86% (A = 30.87%, T = 38.99%, G = 15.54%, and C = 14.60%). The length of 13 PGCs ranges from 159 to 1728 bp. All PCGs initiate with ATG. Nine PCGs terminate with TAA while four PCGs (ATP6, NAD1, NAD4L, and NAD3) terminate with TAG. The length of the 22 *tRNA* genes ranges from 62 to 71 bp. All *tRNA* genes have a conventional cloverleaf shaped secondary structure. The 12S rRNA with a length of 894 bp is located between trnE and trnV while the 16S rRNA with a length of 1421 bp is located between trnV and trnL.

The phylogenetic position of *L. canarium* was further examined based on a maximum-likelihood phylogenetic tree constructed by 13 PCGs in the mitochondrial genome of *L. canarium* and other closely related species using MEGA X (Kumar et al. 2018). The result indicated that *L. canarium* clustered within the Stromboidae (Figure 1).

**Figure 1.**
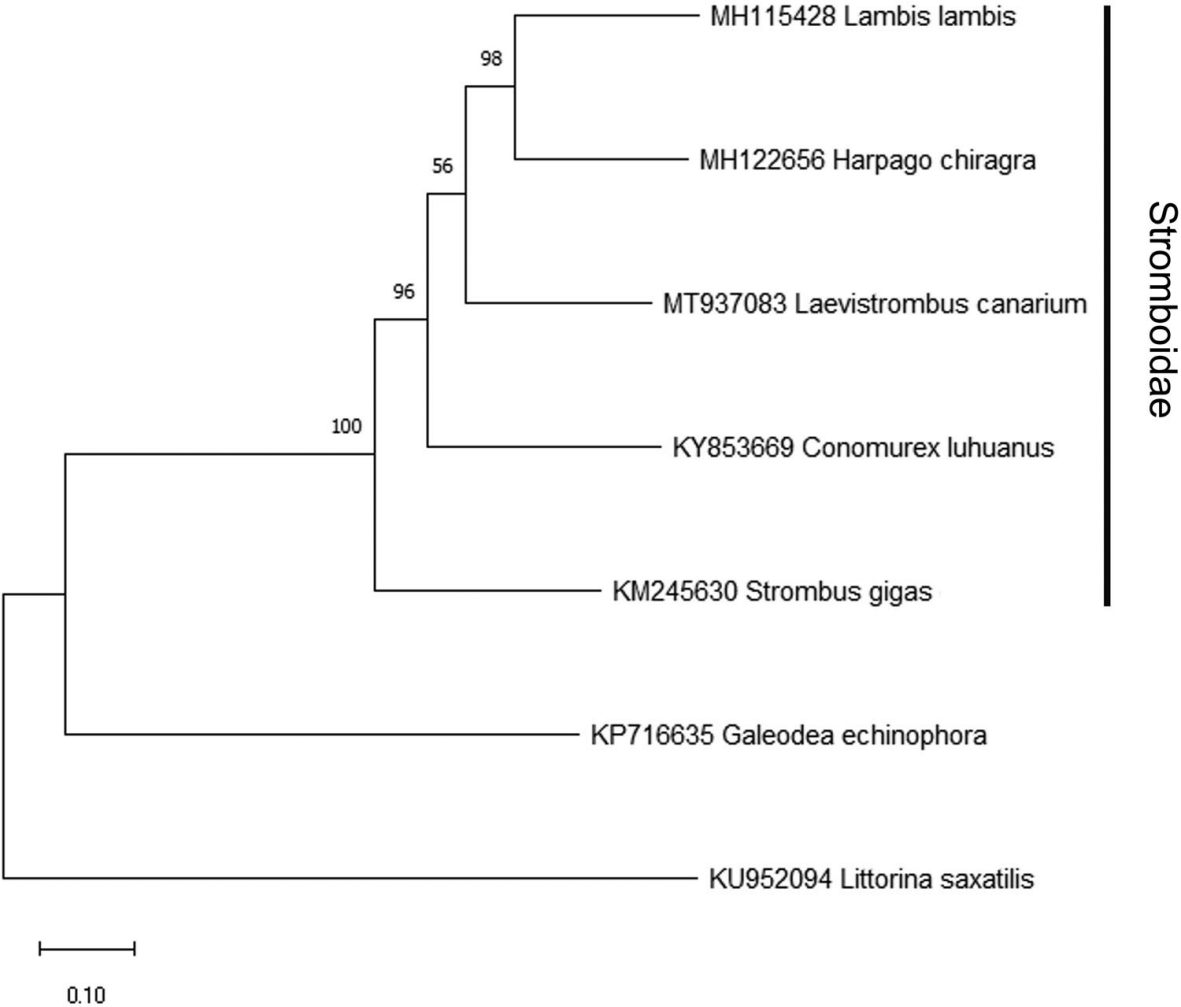
Maximum-likelihood phylogenetic tree constructed by 13 PCGs in the mitochondrial genome of *Laevistrombus canarium* and the other four Stromboidae species. *Galeodae echinophora* and *Littorina saxatilis* are used as the outgroup. Numbers beside each node represent percentages of 1000 bootstrap values.

## Data Availability

The data that support the findings of this study are publicly available in GenBank of NCBI at https://www.ncbi.nlm.nih.gov, accession number MT937083.
